# Chronic administration of the angiotensin type 2 receptor agonist C21 improves insulin sensitivity in C57BL/6 mice.

**DOI:** 10.14814/phy2.13824

**Published:** 2018-08-28

**Authors:** Diego Tomás Quiroga, Marina C. Muñoz, Carolina Gil, Marlies Pffeifer, Jorge E. Toblli, Ulrike M. Steckelings, Jorge F. Giani, Fernando P. Dominici

**Affiliations:** ^1^ Departamento de Química Biológica‐Instituto de Química y Fisicoquímica Biológicas (CONICET) Facultad de Farmacia y Bioquímica Universidad de Buenos Aires Buenos Aires Argentina; ^2^ Laboratory of Experimental Medicine Hospital Alemán de Buenos Aires Buenos Aires Argentina; ^3^ IMM ‐ Deptartment of Cardiovascular & Renal Research University of Southern Denmark Odense Denmark; ^4^ Department of Biomedical Sciences Cedars‐Sinai Medical Center Los Angeles California

**Keywords:** Adiponectin, angiotensin type 2 receptor, compound 21, insulin sensitivity, renin–angiotensin system

## Abstract

The renin–angiotensin system modulates insulin action. Angiotensin type 1 receptor exerts a deleterious effect, whereas the angiotensin type 2 receptor (AT2R) appears to have beneficial effects providing protection against insulin resistance and type 2 diabetes. To further explore the role of the AT2R on insulin action and glucose homeostasis, in this study we administered C57Bl/6 mice with the synthetic agonist of the AT2R C21 for 12 weeks (1 mg/kg per day; ip). Vehicle‐treated animals were used as control. Metabolic parameters, glucose, and insulin tolerance, in vivo insulin signaling in main insulin‐target tissues as well as adipose tissue levels of adiponectin, and TNF‐*α* were assessed. C21‐treated animals displayed decreased glycemia together with unaltered insulinemia, increased insulin sensitivity, and increased glucose tolerance compared to nontreated controls. This was accompanied by a significant decrease in adipocytes size in epididymal adipose tissue and significant increases in both adiponectin and UCP‐1 expression in this tissue. C21‐treated mice showed an increase in both basal Akt and ERK1/2 phosphorylation levels in the liver, and increased insulin‐stimulated Akt activation in adipose tissue. This positive modulation of insulin action induced by C21 appeared not to involve the insulin receptor. In C21‐treated mice, adipose tissue and skeletal muscle became unresponsive to insulin in terms of ERK1/2 phosphorylation levels. Present data show that chronic pharmacological activation of AT2R with C21 increases insulin sensitivity in mice and indicate that the AT2R has a physiological role in the conservation of insulin action.

## Introduction

The renin–angiotensin system (RAS) is an important endocrine system that regulates renal, cardiac, and vascular physiology, either by system effects via the circulation, or via local effects within tissues (Ferrario et al. 2014; Karnik et al. 2015; Carey 2017). Angiotensin (Ang) II is a key hormone in this system. It binds with high affinity to two receptors, the angiotensin type‐1 receptor (AT1R) and the angiotensin type‐2 receptor (AT2R) (Ferrario et al. 2014; Karnik et al. 2015; Carey 2017). Most of the actions of Ang II are mediated by the AT1R (Booz and Baker 1996; Jones et al. 2008; Karnik et al. 2015; Carey 2017; Padia and Carey 2013). The in vivo physiological functions of the AT2R are still not clearly defined, but, in general, it opposes the actions of the AT1R (Padia and Carey 2013; Paulis et al. 2016; Carey 2017). The AT2R is abundantly and ubiquitously expressed in the developing rat fetus and neonatal tissues, where it supports organ development and differentiation. However, its expression declines quickly during the neonatal period showing a relatively low expression compared to AT1Rs in most adult tissues (Ferrario et al. 2014; Karnik et al. 2015; Carey 2017). In fact, the AT2R is expressed at low levels in the normal adult cardiovascular system, adrenal gland, kidney, brain, uterine myometrium, and skin. There are also some examples of tissues that contain more AT2R than AT1R, such as the adrenal medulla, pancreas, and discrete areas of the brain (Ozono et al. 1997; Wang et al. 1998; Allen et al. 1999; Miyata et al. 1999; Padia and Carey 2013; Shao et al. 2013; Karnik et al. 2015; Carey 2017). The AT2 receptor density increases in tissues under pathologic conditions in which inflammation and tissue remodeling occur (Booz and Baker 1996; Lemarié and Schiffrin 2010; Namsolleck et al. 2014; Karnik et al. 2015; Carey 2017). Thus, the modulation of the counterbalance between AT1Rs and AT2Rs has an important therapeutic implication in pathological conditions such as atherosclerosis, insulin resistance, diabetes mellitus, nephropathy, and pulmonary fibrosis (Padia and Carey 2013; Paulis et al. 2016).

Ang II plays a significant role in the development of insulin resistance (Folli et al. 1997; Henriksen 2007; Favre et al. 2015). Chronic Ang II elevation impairs insulin sensitivity and leads to insulin resistance through an AT1R‐dependent mechanism (Folli et al. 1997; Henriksen 2007; Favre et al. 2015). Indeed, AT1R antagonists improve insulin resistance and reduce the incidence of new‐onset diabetes (Folli et al. 1997; Henriksen 2007; Lee et al. 2008b; Shiuchi et al. 2004). However, whether the AT2R actively impacts on insulin sensitivity remains to be elucidated. Pharmacological antagonism of the AT2R with the nonpeptide antagonist PD123319 has indicated a participation of this receptor in insulin sensitivity and glucose homeostasis. Acute infusion of PD123319 has been shown to decrease skeletal muscle glucose uptake in rats (Chai et al. 2010, 2011).

Systemic AT2 receptor blockade reduced the *β*‐cell to *α*‐cell ratio of neonate islets and impaired their insulin secretory function and glucose tolerance (Leung et al. 2014). Recently, we have demonstrated that chronic AT2R blockade with PD123319 reduces the activity of components of main insulin signaling pathways (e.g., insulin receptor and Akt) in mice (Muñoz et al. 2017). The discovery of the selective, nonpeptidic AT2R receptor agonist, Compound 21 (C21), has significantly advanced our current knowledge of AT2R function (Wan et al. 2004). Administration of C21 improved insulin sensitivity in both type 2 diabetic mice and high‐fructose/high‐fat fed rats (Ohshima et al. 2012; Shum et al. 2013). Stimulation of pancreatic AT2R with C21 significantly enhanced insulin synthesis and secretion in adult rats (Nag et al. 2015). Moreover, treatment with C21 prevented streptozotocin induced apoptosis of pancreatic ß‐cells in type 1 diabetic rats (Shao et al. 2014). This beneficial effect on islet cell function and regeneration appears to be via proliferative and antioxidative pathways (Wang et al. 2017). Administration of C21 has recently been shown to enhance insulin delivery and metabolic action in skeletal muscle (Yan et al. 2018). Remarkably, unlike results obtained from AT2R stimulation or antagonism, information obtained from AT2R‐KO mice has not been consistent so far (Shiuchi et al. 2004; Yvan‐Charvet et al. 2005; Samuel et al. 2013; Noll et al. 2015).

In view of available information suggesting a role for the AT2R in metabolism, the aim of the current work was to determine if the AT2R has a physiological role in the modulation of insulin sensitivity and in the control of glucose homeostasis in normal mice. For this, C57BL/6 male mice were treated with daily intraperitoneal injections of C21 for 12 weeks. Metabolic parameters, insulin, and glucose tolerance tests, as well as the status of insulin signaling in main insulin‐target tissues were analyzed. Specifically, we measured the abundance and phosphorylation status of essential insulin signaling components (Boucher et al. 2014), such as the insulin receptor (IR), Akt, and extracellular signal‐regulated kinase (ERK)‐1 and ‐2, in the liver, adipose tissue, and skeletal muscle. We found that C21‐treated animals displayed increased insulin sensitivity and glucose tolerance compared to nontreated controls. This was accompanied by a significant decrease in adipocytes size in epididymal adipose tissue and significant increases in both adiponectin and UCP‐1 expression in this tissue. C21‐treated mice also showed increased insulin‐mediated Akt activation in adipose tissue. Collectively, we provide evidence for an improvement of insulin sensitivity in vivo by chronic AT2R activation through C21, which suggests AT2R stimulation as a possible new therapeutic approach to improve insulin action.

## Materials and Methods

### Study design

All experiments were approved by the Institutional Animal Care and Use Committee of the School of Pharmacy and Biochemistry of the University of Buenos Aires. Twenty‐four male C57BL/6 mice (8 week old) were obtained from School of Veterinary Sciences, National University of La Plata (La Plata, Argentina). Animals were maintained under controlled light and temperature conditions and had free access to water and standard chow diet. To evaluate the effects of chronic AT2R agonism, after 1 week of acclimation (9 weeks of age), animals were randomly distributed into two groups: animals treated with saline (control group) (*n* = 12), and animals treated with C21 (C21 group); (*n* = 12). For 12 weeks, all animals received a daily intraperitoneal (i.p.) injection of either 0.1 mL saline solution (control) or C21 (1 mg/kg; Vicore Pharma AB, Göteborg, Sweden). The dose of C21 was selected based on previously published protocols (Samuel et al. 2013; Nag et al. 2015; Chow et al. 2016) and it is close to the upper limit of therapeutic application of C21. The dose of C21 was adjusted weekly based on body weight changes. On week 11, a glucose tolerance test (GTT) was performed, and after a 2‐day resting period, mice were subjected to an insulin tolerance test (ITT). At the end of the study in week 12, mice were injected with either saline or insulin as described below, euthanized and blood and tissues were collected as described below.

### Glucose and insulin tolerance tests

Mice were fasted for 6 h (8:00 am–14:00 pm) prior to commencement of GTT. Briefly, baseline glucose levels were sampled from tail blood using a commercial glucometer (Accu‐Check,Roche Diagnostics Corp., Indianapolis, IN). Then, mice were injected I.P with 2 g of glucose per kilogram of body weight (BW) using a 20% glucose solution (w/v). and blood glucose was measured at 15, 30, 60, and 120 min postinjection. ITT: Mice were fasted for 6 h, and then injected I.P. with 1 IU porcine insulin (Sigma‐Aldrich) per kilogram of body weight. Blood glucose was measured at 0, 15, 30, 45, 60, and 120 min. The data for both GTT and ITT are presented as absolute values.

### Acute insulin stimulation and tissue collection

After 12 weeks of C21 treatment, mice were fasted for 6 h (8:00 am‐14:00 pm), anesthetized by the i.p. administration of a mixture of ketamine and xylazine (50 and 1 mg/kg, respectively), and submitted to the surgical procedure as soon as anesthesia was assured by the loss of pedal and corneal reflexes. The abdominal cavity was opened, the vena cava was exposed, and a dose of 10 IU porcine insulin/kg body weight dissolved in 0.2 mL of normal saline (0.9% NaCl) was injected via this vein. To obtain data under basal conditions, mice received an injection of vehicle. The liver, adipose tissue (epididymal), and skeletal muscle (hindlimb), were removed after 1, 3, and 6 min, respectively. Serum was obtained from blood of saline‐injected animals by centrifugation (3200*g* for 10 min at 4°C). Tissues and an aliquot of serum (for insulin determination) were kept at −80°C until analysis. Another aliquot of serum was used for determination of circulating triglyceride (TG) and cholesterol concentrations the same day of the sacrifice.

### Tissue homogenization, immunoprecipitation, and immunoblotting

Approximately 500 mg of tissue samples were homogenized in 0.5 mL ice‐cold homogenization buffer containing 1% Triton together with phosphatase and protease inhibitors, as described previously (Muñoz et al. 2017, 2009). Tissue extracts were centrifuged at 100,000*g* for 1 h at 4°C to eliminate insoluble material, and protein concentration in the supernatants was measured using the bicinchoninic acid assay (Pierce BCA Protein Assay Reagent; Thermo Scientific, Waltham, MA). For immunoprecipitation, equal amounts of tissue extracts (2 mg total protein in a final volume of 0.2 mL) were incubated at 4°C overnight with an anti‐IR antibody directed against the IR *β*‐subunit at a final concentration of 4 mg/mL. After incubation, 15 *μ*L protein A‐Sepharose 50% v/v were added to the mixture. Additional samples were incubated in the absence of immunoprecipitating antibody to corroborate that the precipitated proteins were specifically recognized by the immunoprecipitating antibody and not by protein A‐Sepharose. The preparation was further incubated with constant rocking for 2 h at 4°C and then centrifuged at 1000*g* for 30 sec. The supernatant was discarded and the precipitate was washed three times with buffer containing 0.05 mol/L Tris, 0.01 mol/L sodium vanadate, and 1% w/v Triton X‐100 (pH 7.4). The final pellet was resuspended in 50 *μ*L Laemmli buffer, boiled for 5 min, and stored at 20°C until electrophoresis. Briefly, equal amounts of solubilized protein (40 *μ*g) were separated by SDS‐PAGE. Proteins were then transferred to PVDF membranes and blotted with antiphospho‐IR, antiphospho‐Akt, and antiphospho‐Erk1/2 antibodies (1:2000 dilution for all antibodies). The abundance of adiponectin and TNF‐*α* in adipose tissue was detected in tissue lysates using specific antibodies at 1:2000 dilution; GAPDH was used as a loading control at a 1:10,000 dilution. Immunoblotting was performed according to previously described protocols (Muñoz et al. 2009). Imunoprecipitated IR samples were subjected to immunoblotting with either a specific antiphosphotyrosine or with the same antibody used for immunoprecipitation to determine protein abundance of the IR. The abundance of Akt, and ERK1/2 was detected in tissue lysates using the corresponding antiprotein antibodies at 1:2000 dilution. After extensive washing, membranes were incubated with the appropriate secondary HRP‐coupled antibody at 1:20,000 dilution and processed for enhanced chemiluminescence (ECL) using the Pierce ECL plus Western blotting detection system (ThermoFisher Scientific, Waltham, MA). Bands were quantified using Gel‐Pro Analyzer 4.0 (Media Cybernetics, Bethesda, MD). The content of GADPDH in both immunoprecipitated and solubilized protein extracts was used as a loading control and was assessed in independent inmunoblottings using the same volume of samples used for determination for analysis of the phosphorylation and content of insulin signaling molecules.

### Assessment of blood chemistry

Plasma cholesterol and triglycerides were measured with enzymatic colorimetric assay kits (Wiener Lab, Rosario, Argentina). Serum insulin levels were assessed using a mouse insulin ELISA kit (Crystal Chem, Downers Grove, IL).

### Immunohistochemistry

Portions of epididymal adipose tissue of each animal were fixed in phosphate‐buffered 10% formaldehyde (pH 7.2) and embedded in paraffin. Three micrometer sections were cut and processed for immunohistochemical study. Immunolabeling of specimens was carried out with a modified avidin–biotin–peroxidase complex technique using a Vectastain ABC kit (Universal Elite; Vector Laboratories, Burlingame, CA). Following deparaffinization and rehydration, sections were washed in PBS for 5 min. Quenching of endogenous peroxidase activity was achieved by incubating the sections for 30 min in 1% hydrogen peroxide in methanol. After being washed in PBS (pH 7.2) for 20 min, they were incubated with blocking serum for 20 min. The sections were then incubated with the primary antibody, rinsed in PBS, and incubated with biotinylated universal antibody for 30 min. After being washed in PBS, the specimens were incubated for 40 min with Vectastain Elite ABC reagent (Vector Laboratories, Burlingame, CA) and exposed for 5 min to 0.1% diaminobenzidine (Polysciences, Warrington, PA) and 0.2% hydrogen peroxide in 50 mmol/L Tris buffer, pH 8. Antibodies against adiponectin, TNF‐*α* and nitrotyrosine were used at a 1:200 dilution, the antibody against UCP‐1 was used at 1:500 dilution. All observations were performed using light microscopy (E400; Nikon Instruments, Melville, NY) at a ×10 magnification, and histological images were captured with a digital camera and processed by an image analyzer (Image‐Pro Plus 4.5 for Windows; Media Cybernetics, Silver Spring, MD). Immunolocalization was calculated by computer for adipose tissue using the image analyzer and expressed as percentage of the area with positive staining for adiponectin, nitrotyrosine‐containing proteins, TNF‐*α,* and UCP‐1. In all cases, two independent observers performed a blinded evaluation, and the mean percentage value was then calculated.

### Morphological analysis of adipose tissue

Epididymal adipose tissue samples, preserved in buffered formalin solution, were used to prepare 3‐*μ*m‐thick paraffin‐embedded sections that were deparaffinized in xylene and rehydrated to water through ethanol at 100°, 96°, and 80°C (Muñoz et al. 2009). For each individual sample, adipocyte size was measured in four microscopic fields separated by at least 100 *μ*mol/L (in single‐blinded condition). The size of 200 adipocytes was determined for each mouse. Images were acquired using a Nikon E400 microscope at x10 magnification after hematoxylin‐eosin (H & E) staining. The adipocyte diameter was expressed in *μ*m.

### Materials and reagents

The rabbit polyclonal antiinsulin receptor (IR) *β*‐subunit (C19; sc‐711), the mouse monoclonal antiphosphotyrosine (PY99; sc‐7020), the goat polyclonal anti‐rabbit IgG conjugated with HRP (sc‐2004), the goat anti‐mouse IgG‐HRP (sc‐2005), and rabbit anti‐goat IgG‐HRP (sc‐2768) antibodies were purchased from Santa Cruz Biotechnology, Inc. (Santa Cruz, CA). The rabbit polyclonal antibody antiphospho‐Akt‐S473 (4060), the rabbit polyclonal antibody antiphospho‐Akt‐T308 (9275), the anti‐Akt (pan) rabbit monoclonal antibody (C67E7) that detects endogenous levels of total AKT1, AKT2, and AKT3 protein, the rabbit monoclonal, the rabbit monoclonal antibody antiphospho‐ERK1/2, that detects ERK‐1 and ‐2 (p44 and p42 MAPK, respectively) when phosphorylated at T202 and Y204 (4370) and the rabbit polyclonal antibody anti‐ERK1/2 (9102) were from Cell Signalling (Danvers, MA). The goat polyclonal anti‐mouse TNF‐*α* (MAB4101) and antiadiponectin (AF1119) antibodies were from R&D systems (Minneapolis, MN). The rabbit polyclonal antibody antinitrotyrosine (AB5411) was from Millipore Sigma (St. Louis, MO). The rabbit polyclonal antibody anti‐UCP‐1 (PA1‐24894 was from Thermo Fisher (Rockford, IL). Protein loading in the gels was evaluated with a mouse monoclonal anti‐GADPDH (ab9485) from Abcam (San Francisco, CA).

### Statistical analyses

Values are reported as means ± SEM unless specified otherwise. Statistical significance was determined by *t* test when two groups were compared (metabolic parameters), by one‐way ANOVA (GTT and ITT) or by two‐way ANOVA, followed by Tukey′s multiple comparison post hoc test when multiple groups were compared. We assigned significance at *P* < 0.05. In all the figures, * *P* < 0.05, ** *P* < 0.01. All analyses were performed using GraphPad Prism 6 (GraphPad Software, San Diego, CA).

## Results

### C21‐treatment improves insulin sensitivity and glucose tolerance in male C57Bl/6 mice

**Figure 1:**
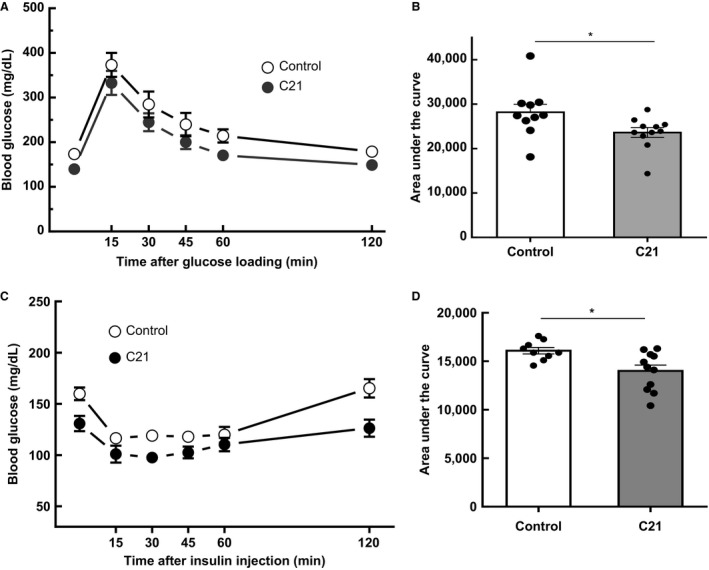
Chronic administration of compound 21 improves glucose tolerance and insulin sensitivity. (A) GTT. (B) calculated area under the curve from the GTT in control and C21‐treated mice. (C) insulin tolerance test (ITT). (D) calculated area under the curve from the ITT in control and C21‐treated mice. Mice were fasted for 6 h prior to basal glucose monitoring (as described in Materials and Methods) and subsequently injected i.p. with a 20% glucose solution (w/v). Blood was reanalyzed at 15, 30, 45, 60, and 120 min postinjection (C21, *n* = 12 per group; control, *n* = 10 per group. Data are represented as mean ± SEM. and analyzed by one‐way ANOVA followed by Tukey′s multiple comparison *t* tests where *P <* *0.05 vs. control mice. GTT, glucose tolerance test.

Treatment with C21 did not modify body weight of the animals. Body weight of the mice was 28 ± 2 g at treatment start and 31 ± 1 g at the end of treatment in both, the vehicle and the C21‐treated groups of animals (Table 1). Remarkably, blood glucose levels were significantly lower in mice treated chronically with C21 (20 ± 2% reduction; *P *<* *0.01 versus control values). This finding is in accordance with the observation that C21‐treated mice displayed improved glucose handling as shown in a GTT (Fig. 1A and B). The area under the curve for the GTT was significantly lower in C21‐treated mice (Fig. 1B; *P *<* *0.05 vs. the control group). These changes were associated with augmented insulin sensitivity in C57Bl/6 mice (Fig. 1C and D). The area under the curve for the ITT was significantly lower in C21‐treated mice compared to the saline‐treated control group (Fig. 1D; *P *=* *0.0106 vs. nontreated control animals). The beneficial change in glucose handling in the C21‐treated group coincided with unaltered circulating levels of insulin and triglycerides and with a marginal but although not significant decrease in the levels of cholesterol compared to control.

**Table 1:** Anthropometric and biochemical markers of the experimental animals

Parameter	Control	C21
Body weight, g		
Beginning of study	28 ± 2 (12)	28 ± 2 (12)
End of study	32 ± 1 (10)	32 ± 1 (11)
Glucose, mg/dL	169 ± 23 (10)	138 ± 19 (12)^a^
Insulin, ng/mL	0.4 ± 0.09 (5)	0.43 ± 0.07 (6)
Triglycerides, mg/dL	46 ± 4(5)	42 ± 5 (6)
Cholesterol, mg/dL	72 ± 3(5)	59 ± 4 (6)
Epididymal adipose tissue weight, g	0.42 ± 0.03 (10)	0.34 ± 0.04 (12)

Values are means ± SD. The number of samples used for each determination (*n*) is shown in parentheses.

a *P *<* *0.01 when compared with control mice.

### C21‐treatment induces changes in the insulin signaling pathway downstream of the insulin receptor in insulin‐target tissues of C57Bl/6 mice in vivo

**Figure 2:**
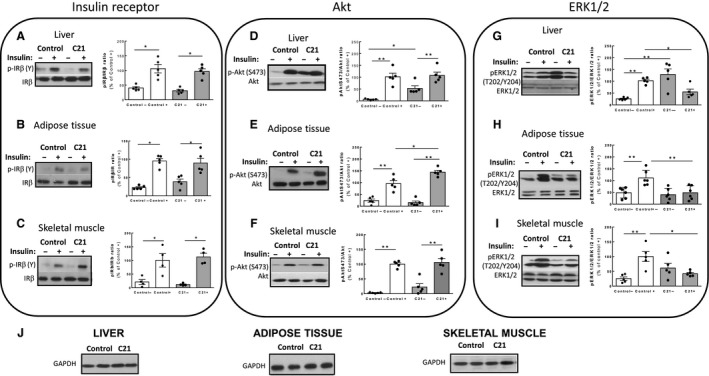
Chronic administration of compound 21 modulates liver, adipose tissue, and skeletal muscle insulin signaling. Western blot analysis of tissue from control saline‐treated and C21‐treated mice after acute injection with insulin (+) or vehicle (−). (A–C) Phosphorylation levels of the IR *β*‐subunit at tyrosine (Y) residues in were determined in immunoprecipitated samples using antiphosphotyrosine antibodies as described in Materials and Methods. D–F: Specific antibodies were used to detect phosphorylation of Akt at Serine (S) 473. (G–I) Phosphorylation of ERK1/2 at activating residues Threonine (T) 202 and Tyrosine (Y) 204; (C21, *n *=* *4–5 per group; control, *n *=* *4–5 per group). Bar graphs represent the quantification of phosphorylated (p) IR at tyrosine (Y) residues, Akt at Ser473 (S473) and ERK1/2 at Threonine 202 and Tyrosine 204 (T202/Y204) related to the abundance of the corresponding protein as measured with anti‐IR *β*, anti‐Akt, and anti‐ERK1/2 antibodies. (J) GADPH abundance was measured as a loading control in the same samples. Data are represented as mean ± SEM and analyzed by two‐way ANOVA followed by Tukey′s test where *P <* *0.05 and **P <* *0.01 when compared with the group connected by a line. IR, insulin receptor.

To investigate the possible mechanism of the C21‐mediated improvement of glucose tolerance and insulin sensitivity, we analyzed the in vivo status of main insulin signaling components in insulin‐target tissue of treated mice. As expected, insulin itself let to a significant increase in the phosphorylation of the insulin receptor, Akt and ERK1/2 in liver, adipose tissue and skeletal muscle (Fig. 2A–I). Basal‐ or insulin‐induced insulin receptor phosphorylation levels were not altered by C21 treatment in all three organs (Fig. 2A*–*C). In the liver, treatment with C21 induced a significant increase in the basal, but not in the insulin‐induced phosphorylation of Akt at Ser473 when compared with control mice (Fig. 2D; *P *<* *0.01), In contrast, in adipose tissue, basal phosphorylation of Akt at Ser473 was unchanged by C21 treatment, but the insulin‐induced phosphorylation levels of this kinase at Ser473 were significantly increased by AT2R stimulation when compared with insulin‐treated control mice (49 ± 7% increase, *P* < 0.05; Fig. 2E). Notably, in skeletal muscle of C57Bl/6 mice, phosphorylation of Akt at Ser473 remained unaltered after C21 treatment both in basal conditions and after insulin stimulation (Fig. 2F). Treatment with C21 induced a tissue‐specific modulation of ERK1/2 phosphorylation. Basal phosphorylation levels of ERK1/2 at Thr202 and Tyr204, indicative of activation of these enzymes, were markedly increased in liver of C21‐treated mice when compared with control mice (approximately 4‐fold increase; *P* < 0.01; Fig. 2G), but not in adipose tissue and skeletal muscle (Fig. 2H and I). In contrast, C21 administration inhibited the insulin‐mediated phosphorylation of ERK1/2 in all analyzed tissues. (Fig. 2G*–*I).

### Treatment with C21 decreases adipocyte size in C57Bl6/mice

**Figure 3:**
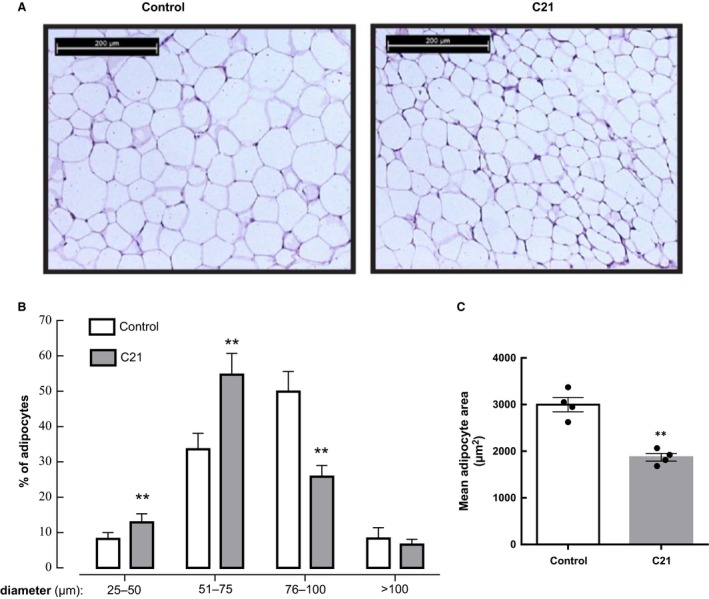
Chronic administration of compound 21 decreases adipocyte size. Epididymal adipose tissue was prepared for histology. Paraffin sections (3 *μ*m) were stained with hematoxylin and eosin and images acquired as described in Materials and Methods section. (A) Representative histology images were obtained in adipose tissue sections at x10 magnification. (B) Adipocyte size distribution in control and C21‐treated mice. The cell size of 200 adipocytes was measured in four different microscopic fields for each sample, and the average was used as the value of each sample (C21, *n *=* *4 per group; control, *n *=* *4 per group). Data are represented as mean ± SEM. and analyzed by two‐way ANOVA followed by Tukey′s multiple comparison *t* tests where ** *P* < 0.01 when compared with the respective control diameter range. (C) Adipocyte area was calculated from adipocyte diameters measured in panel B (C21, *n *=* *4 per group; control, *n *=* *4 per group). Data are presented as means ± SEM and analyzed by unpaired two‐tailed Student's *t* test where ** *P *<* *0.01 when compared with control mice.

As shown in Table 1, C21 treatment induced an apparent decrease in absolute epididymal adipose tissue weight, but this change was not statistically significant (*P *=* *0.138). As shown in Figure 3A and C, the mean area of adipocytes from epididymal fat was significantly lower in mice treated with C21 (1858 ± 79 *μ*m^2^) when compared with control mice (2999 ± 152 *μ*m^2^). The proportion of cells in the 25–50 *μ*m and 51–75 *μ*m diameter range was higher in adipose tissue from C21‐treated mice that in control mice (*P *<* *0.01; Fig. 3B). In contrast, C21‐treated mice showed a lower proportion of cells in the 76–100 *μ*m size; Fig. 3B). No significant changes were observed between C21‐treated mice and control mice in the highest size range analyzed (>100 *μ*m), but the number of these cells was generally very low.

### The enhanced insulin sensitivity of C21‐treated mice is accompanied by increased abundance of adiponectin and UCP‐1 in white adipose tissue

**Figure 4:**
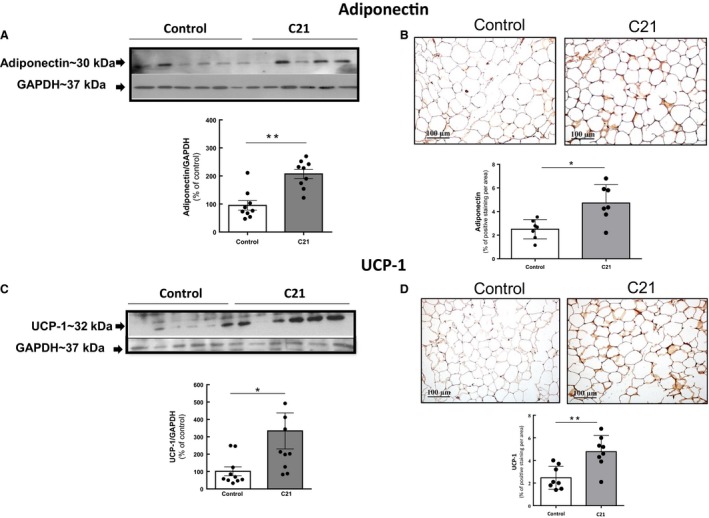
Chronic administration of compound 21 increases the abundance of adiponectin and UCP‐1 in adipose tissue. (A) and (C) Abundance of adiponectin and UCP‐1 in adipose tissue as determined by western blotting. (B) and (D) Levels of adiponectin and UCP‐1 as determined by immunohistochemistry. Quantification of specific bands was performed with Gel‐Pro Analyzer software. (A) and (C) Scatter dot plots show individual values and superimposed bar graphs represent the means ± S.E. (B) and (D) Positive adiponectin and UCP‐1 staining was quantified using Image‐Pro Plus 4.5 software. Data were analyzed by unpaired two‐tailed Student's *t* test, where **P <* *0.01 when compared with saline‐treated control mice. For adiponectin, (C21, *n *=* *9 per group; control, *n *=* *9 per group). For UCP‐1, (C21, *n *=* *9 per group, control, *n *=* *10 per group).

**Figure 5:**
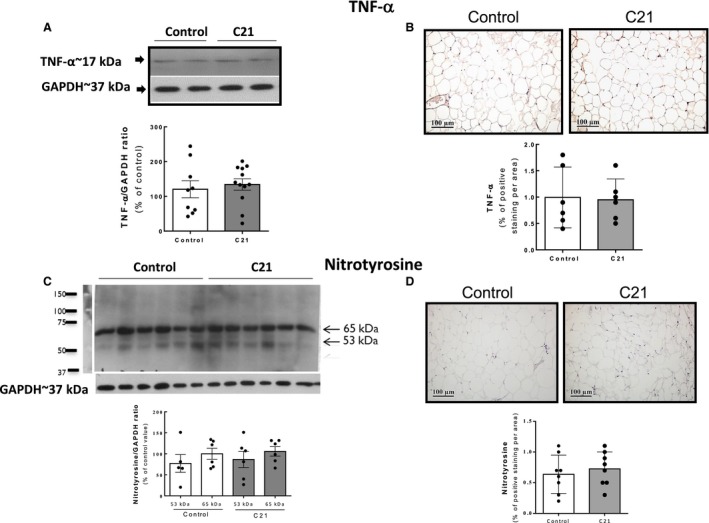
Parameters of inflammation/nitrosative stress are unaltered in adipose tissue after chronic administration of compound 21. (A) and (B) Levels of TNF‐*α* determined by WB and IHC. (C) and (D) levels of nitrotyrosine determined by WB and IHC. Quantification of specific bands was performed with Gel‐Pro Analyzer software. (C) For nitrotyrosine quantification, the intensity of the most predominant bands in the WB (of unknown identity), with MWs of 53 and 65 kDa, respectively, were quantified in each sample. Bar graphs are the means ± SE. (B) and (D) Positive TNF‐*α* and nitrotyrosine staining as detected by immunohistochemistry was quantified using Image‐Pro Plus software. Data were analyzed by unpaired two‐tailed Student's *t* test. C21, *n *=* *9 per group, control, *n *=* *12 per group for TNF‐*α* quantification. For nitrotyrosine quantification by Western blotting: C21, *n *=* *6 per group; control, *n *=* *6 per group and by immunohistochemistry: C21, *n *=* *9 per group; control, *n *=* *9 per group. WB, western blotting.

Given that the enhanced insulin sensitivity and glucose tolerance observed after C21 treatment was associated with decreased adipocyte size, we performed further evaluation of adipose tissue status. Treatment with C21 induced a significant increase in adiponectin abundance in epididymal adipose tissue assessed by both western blotting (WB) and IHC (approximately twofold; *P* < 0.01 vs. control values); (Fig. 4A and B). This finding was consistent with a significant increase in UCP‐1 abundance in this tissue (approximately 3‐fold; *P *<* *0.01 vs. control values Fig. 4C and D). There was no significant difference in the content of the proinflammatory cytokine TNF‐*α* in adipose tissue between the two experimental groups (Fig. 5A and B), Nitrotyrosine levels in adipose tissue proteins were measured as a parameter of oxidative/nitrosative stress. The levels of proteins nitrated at Tyr residues were similar between C21‐treated and saline‐injected controls (Fig. 5C and D).

## Discussion

Our findings show that C57BL/6 mice exposed to chronic, systemic pharmacological AT2R agonism with C21 display decreased glycemia, enhanced glucose tolerance, and improved insulin sensitivity, suggesting an active role of the AT2R in the modulation of glycemic control under physiological conditions. We present evidence that this improvement of glucose homeostasis is associated with an activation of Akt and ERK1/2 under basal conditions in the liver and with an enhancement of insulin‐induced Akt activation in adipose tissue. Concomitantly, C21 treatment led to a blunted insulin response as indicated by an inhibition of insulin‐induced ERK1/2 activation in both skeletal muscle and adipose tissue. Adipose tissue appears as a major target of AT2R agonism and, presumably, mediates many of the positive metabolic changes induced by C21. Specifically, C21 treatment was associated with decreased adipocyte size, increased levels of the insulin‐sensitizing adipokine adiponectin and augmented UCP‐1 levels, a marker of adipose tissue browning.

Previous reports have shown an improvement of insulin action and amelioration of insulin resistance after C21 treatment in different rodent models of insulin resistance and type 2 diabetes, including KK‐Ay diabetic mice (Ohshima et al. 2012), high‐fat/high‐fructose fed rats (Shum et al. 2013), STZ‐diabetic adult and neonatal rats (Shao et al. 2014; Wang et al. 2017), and Zucker diabetic fatty rats (Castoldi et al. 2014). Our results are consistent with these results and particularly, with those obtained in normal male Sprague–Dawley rats where C21 administration increased glucose tolerance and insulin sensitivity through an apparent insulinotropic effect (Shao et al. 2014). In line with these findings, our current results indicate that AT2R modulates insulin action under physiological conditions and reinforce the notion that AT2 agonism might have therapeutic potential in the management of diabetes and its associated complications.

It has been previously shown that chronic treatment with AT2R agonists reduces the size of hypertrophic adipocytes and adipose tissue mass in rodents fed with high‐fat diet (Shum et al. 2013), suggesting the favorable effects of AT2R activation for obesity. We showed that treatment with C21 was associated with decreased adipocyte area, resulting from decreased number of large adipocytes and increased number of new, small adipocytes. In humans, adipose cell enlargement, in association with decreased expression of lipogenic genes, and expansion of visceral adipose tissue and intrahepatic fat are mediators of obesity‐related insulin resistance (Salans et al. 1968; McLaughlin et al. 2016). Thus, the reduction in adipocyte size in C21‐treated mice could be considered as an additional parameter of enhanced insulin action originating from AT2R activation.

Angiotensin II through the AT1R, has been shown to attenuate Akt activity through an ERK1/2‐dependent mechanism (Dünner et al. 2013). Interestingly, our current results indicate that C21 increases insulin‐stimulated activation of Akt and simultaneously inhibits insulin‐stimulated ERK1/2 activation in adipose tissue. These data are in accordance with a recent study performed in isolated mouse adipocytes showing that C21 increases Akt and AMPK activation through a mechanism that might involve attenuation of ERK1/2 phosphorylation (Than et al. 2017). The enhancement of insulin‐stimulated Akt activation in adipose tissue might contribute to the ability of C21 to enhance glucose uptake in this tissue (Ohshima et al. 2012). Noteworthy, chronic blockade of the AT2R by administration of PD123319 resulted in decreased insulin signaling in liver and adipose tissue but did not modify the status of the insulin signaling system in skeletal muscle (Muñoz et al. 2017). Our current results and previous findings suggest that glucose handling of the skeletal muscle is unaltered after modulation (antagonism or agonist) of the AT2R in healthy animals in vivo. This could be ascribed to the low levels of AT2R compared to AT1R present in this tissue. This situation might change during pathological conditions where AT2R largely such as skeletal muscle injury (Painemal et al. 2013) and also in diabetic states (Lee et al. 2008a). In this aspect, our studies do not agree with a recent report performed in rats, showing that a short‐term C21 infusion enhances insulin delivery and action in muscle (Yan et al. 2018). This could be ascribed to the several factors, including differences in the duration of the experimental protocol as well as the dose employed.

Regarding the mechanism by which AT2R agonist inhibits insulin‐induced phosphorylation of ERK1/2, it is well established that the regulatory dephosphorylation of ERK1/2 is mediated by protein‐tyrosine‐specific phosphatases, protein‐serine/threonine phosphatases, and dual specificity phosphatases (Roskoski 2012). Phosphatase activation has been one of the first signals associated with AT2R activation (Guimond and Gallo‐Payet 2012; Carey 2017). MKP‐1, is a key regulator of ERK1/2 activity. AT2R‐activated MKP‐1 has been observed in various cell types (Guimond and Gallo‐Payet 2012). Activation of MKP‐1 by AT2R leads to a decrease in ERK1/2 activity (Guimond and Gallo‐Payet 2012), indicating that this could be a potential mechanism by which C21 treatment could lead to attenuation of insulin‐induced ERK1/2 activation.

The increase in basal activation of ERK1/2 in the liver is unexpected and might be the result of a direct action of C21 in the hepatocytes. In previous studies performed in neurite cells in vitro and in mouse heart in vivo, C21 incubation or administration has been shown to stimulate ERK1/2 phosphorylation (Wan et al. 2004; Kaschina et al. 2008). Thus, it is possible that prolonged stimulation of the AT2R might result in increased basal activation of ERK1/2. It remains to be determined whether this change is linked to the enhanced insulin sensitivity observed in C21‐treated animals or it is unrelated to this modification.

AT2Rs are expressed at low levels in healthy tissues during physiologically quiescent states (Jones et al. 2008; Padia and Carey 2013; Carey 2017). However, in the current study, C21 exerted a positive modulation of insulin action on normal mice indicating that these physiological levels of AT2R are functional and capable of inducing a metabolic response. Given that the affinity of C21 for the AT2R is 25 000‐fold higher than for the AT1R, there is a strong indication that the insulin‐sensitizing effects of C21 are specifically mediated through the AT2R (Wan et al. 2004).

Adiponectin is a major regulator of glucose and lipid homeostasis via its insulin‐sensitizing properties, and the reduction in this adipokine is associated with the development of metabolic syndrome and type 2 diabetes (Fang and Sweeney 2006). Administration of adiponectin has been shown to lower plasma glucose levels by improving insulin action in mice (Berg et al. 2001; Kubota et al. 2002). Thus, the increase in adiponectin levels in adipose tissue after treatment with C21 could be an important contributor to the enhancement of insulin action observed. Treatment with C21 has been reported to increased serum adiponectin levels both in diet‐induced insulin‐resistant rats (Shum et al. 2013) and in normal mice (Than et al. 2017).

UCP‐1 is a brown adipocyte‐specific protein with a key role in the thermogenic process. It promotes mitochondrial respiration and heat generation by decreasing the proton gradient generated in oxidative phosphorylation, hence the mitochondrial membrane potential (Krauss et al. 2005). It has been shown that both AT1R antagonism (Tsukuda et al. 2016) and incubation with C21 (Than et al. 2017) increase UCP‐1 levels in mouse adipocytes and that in the presence of an AT1R blocker, angiotensin II through AT2R mimics this upregulating effect (Than et al. 2017). We found that UCP‐1 levels are significantly increased in mouse adipose tissue after chronic administration of C21 reinforcing the notion that AT2R agonism can induce adipocyte browning. Given the importance of the browning of adipose tissue in regulating energy metabolism and its therapeutic implication for the treatment of obesity, AT2R modulation appears as a very attractive therapeutic approach to treat obesity, insulin resistance, and metabolic syndrome‐like states.

### Perspectives and significance

Prolonged treatment with the AT2R agonist C21 reduces blood glucose levels and enhances insulin sensitivity and glucose tolerance in C57Bl/6 mice. These beneficial effects were not related to an increase in insulin production since circulating insulin levels remained unaltered after treatment with C21. Interestingly, a potential molecular mechanism behind the beneficial effects of C21 could be the modulation of the in vivo insulin signaling of adipose tissue as shown by an improved response to insulin in terms of Akt phosphorylation associated with a drastic attenuation of insulin‐stimulated ERK1/2 activation. We also observed that changes in insulin signaling were tissue‐specific: while insulin signaling was mainly unaltered in skeletal muscle, the liver of C21‐treated mice showed increased basal activation of both Akt and ERK1/2. Altered insulin signaling in the liver indicates a potential participation of this tissue in enhanced insulin action, presumably, involving inhibition of gluconeogenesis. However, this hypothesis requires further exploration. Modulation of adipose tissue function should also be considered an important contributor to the observed metabolic changes. Both adiponectin and uncoupling protein‐1 levels were upregulated in adipose tissue after the chronic agonism of the AT2R. Thus, chronic AT2R agonism improves glucose homeostasis and supports the therapeutic potential of AT2R modulation in metabolic diseases.

## Conflict of Interest

UMS received modest research support (free drug supply; financial support for attending conferences) from Vicore Pharma, which is the company owning C21. UMS is currently employed part‐time by Vicore Pharma, but has not been employed during the conduct of the study and the analysis of data.
